# Perception of aesthetic procedures applied to the vulva in the context of the medical specialty of gynecology and obstetrics

**DOI:** 10.1093/sexmed/qfag003

**Published:** 2026-02-16

**Authors:** Vera Lúcia Mota da Fonseca, Jorge Artur Peçanha de Miranda Coelho, Gustavo Henrique Silva de Souza, Emanuel Duarte de Almeida Cordeiro, José Humberto Belmino Chaves, Rui Manuel Lopes Nunes

**Affiliations:** Faculty of Medicine, University of Porto, Porto, 4200-319, Portugal; Faculty of Medicine, Federal University of Alagoas, Maceió, AL 57072-900, Brazil; Department of Management, Federal Institute of Northern Minas Gerais, Teófilo Otoni, MG 39803-294, Brazil; Faculty of Psychology, State University of Southwest Bahia, Vitória da Conquista, BA 45083-900, Brazil; Faculty of Medicine, Federal University of Alagoas, Maceió, AL 57072-900, Brazil; Faculty of Medicine, University of Porto, Porto, 4200-319, Portugal

**Keywords:** sexual health, labiaplasty, medical perception, medical ethics

## Abstract

**Background:**

Female genital cosmetic surgery (FGCS), particularly labiaplasty, has gained increasing visibility and demand in clinical practice.

**Aim:**

To examine the perceptions of gynecology physicians and residents regarding ethical aspects and knowledge related to FGCS, with a focus on labiaplasty.

**Methods:**

A cross-sectional study was conducted with 404 physicians, including 327 (80.9%) women and 77 (19.1%) men. Participants completed a structured questionnaire assessing their ethical perspectives on vulvovaginal cosmetic procedures and their specific knowledge of interventions such as labiaplasty, hymenoplasty, clitoroplasty, and others.

**Outcomes:**

Differences in ethical views and knowledge levels between experienced physicians and residents were evaluated.

**Results:**

The findings revealed no significant differences between physicians and residents in ethical perceptions of labiaplasty. However, more experienced physicians reported greater exposure to patients seeking these procedures and demonstrated higher levels of knowledge regarding specific techniques.

**Clinical Implications:**

Despite greater clinical exposure, ethical perspectives seem to be shaped early during academic training, underscoring the importance of formal education on FGCS.

**Strengths and Limitations:**

This study provides insights into a relatively underexplored topic in medical education. However, the use of self-reported measures and a cross-sectional design may limit causal interpretations.

**Conclusion:**

Ethical perceptions of FGCS are consistent across levels of clinical experience, suggesting a strong influence of medical training and reinforcing the need for structured discussions on the topic in educational curricula.

## Introduction

A surgical procedure called labiaplasty is used to shrink the labia minora.^1^ The use of labiaplasty growing in popularity in recent years. For instance, 18 813 labiaplasty surgeries were recorded in the United States in 2021, a 36% increase over 2020.^1^ The number of labiaplasties performed increased by 14.8% from 164 667 in 2019 to 189 058 in 2023, according to the International Society of Aesthetic Plastic Surgery.^2^

While vaginal repair procedures, such as the correction of fistulas and prolapses, have a long history in gynecology, the idea of surgical labial alteration for aesthetic purposes is relatively new. The first reports of cosmetic labiaplasty emerged in 1984. From the late 1990s and early 2000s, the concept of the “designer vagina” entered public discourse, driven by clinical reports and extensive media coverage, including prominent women’s magazines and the presence of surgeons on websites. Since then, case studies and clinical commentaries have addressed different techniques for labial reduction.^3^

More recent findings attribute an increase in the search for these procedures to an idealized perception of what would be perfect, as seen in pornographic materials and feminine symbols. This vision, often unattainable, reinforces the importance of health professionals understanding the motivations behind this demand, distinguishing them from issues of physical and psychological health.^4^

The increasing demand for these interventions raises questions about the motivations that drive them, which are often based on misguided beliefs about what constitutes “normal” genital anatomy. Therefore, it is essential for gynecologists to be trained to identify signs of psychological disorders, such as body dysmorphic disorder, that may drive the demand for these procedures. Recognizing signs related to depression and anxiety can significantly contribute to appropriate referrals, helping patients address the root psychological issue and potentially preventing substantial risks associated with the labiaplasty procedure.^5–7^

Professionals must critically consider ethical principles, such as autonomy, beneficence, non-maleficence, justice, and truthfulness, when evaluating patients. Adherence to the principle of non-maleficence is particularly critical, given that the labiaplasty procedure carries risks, and women may experience unexpected sequelae or mutilations in their genital organs. This necessity to avoid harm underscores the importance of a comprehensive approach that prioritizes psychological screening before intervention.^8^

Even though 80%-90% subjective success rates have been reported, these studies are frequently constrained by their retrospective design, brief follow-up periods, cursory handling of body image issues, and absence of control groups.^9^ When analyzing the methodological aspects that support the indicators of success, it becomes evident that these results are primarily based on patient satisfaction. This reliance presents limitations within the medical literature, as subjective perception does not necessarily correspond to the effectiveness of the procedure or to positive long-term outcomes for the patient. Therefore, it is essential to emphasize the importance of studies that employ alternative methodological approaches to identify the actual impacts of female genital surgeries. In this regard, conducting interviews with professionals and patients within this context can contribute to a deeper understanding of the perspectives involved.^10^ From interviews with professionals, it was identified that the reasons given by women for seeking female genital cosmetic surgery (FGCS) were public depilation, media representation, pornography, advertising regulations, social pressure, and genital unfamiliarity. Ethical perspectives were also problematic, and there was discursive contradiction surrounding diversity and the normal vulva.^10^^,^^11^

The ideal of hairless, “neat” vulvas is promoted by the media, especially pornography, which increases social pressure on women to fit in.^12^ The pornographic environment that can be seen on specialized websites on the internet is an example of unrealistic bodily harm, or standards that are not met outside of that context.^10^ This setting, along with the cosmetic surgery industry’s relentless marketing, fosters a market that pushes women to have irreversible procedures done in order to satisfy limited notions of beauty.^12^ Furthermore, women are under more pressure to adhere to particular vulvar aesthetics due to heteronormative and androcentric representations of sexual pleasure that place a higher priority on heterosexual vaginal sex.^13–15^

Within this context, it is essential to conduct investigations that explore the perceptions of both professionals and patients regarding female intimate surgeries. Such studies should aim to understand the motivations behind professionals’ recommendations for these interventions, taking into account ethical considerations as well as the potential benefits and impacts of these procedures. The issues in this scenario suggest that the performance of these surgeries is not free from professional conflicts, as there is a recognition of women’s rights over their bodies. However, there remains a concern regarding the underlying motivations driving these decisions.^10^

This research consists of a study employing an observational design with a convenience sample of 404 participants, including gynecologists-obstetricians and residents. The study focuses on their ethical perspectives regarding FGCS. Data were collected through online questionnaires, and statistical analyses were conducted to compare the opinions of gynecologists-obstetricians with those of residents.

There is a growing demand for vulvar aesthetic procedures, accompanied by an ongoing debate about their medical, psychological, and ethical implications. Understanding how gynecologists and obstetricians perceive these interventions is therefore essential. This study aims to explore physicians’ ethical perceptions, knowledge levels, and the influence of clinical experience regarding vulvar aesthetic procedures. To address this aim, we state the following hypothesis:

**H1**: Considering these considerations, this study aims to explore the perceptions of medical professionals regarding vulvar aesthetic procedures. To address this aim, we seek to answer the following research question: Are there no significant differences in the ethical perception of vulvar aesthetic procedures between experienced gynecologists-obstetricians and residents, and do experienced physicians demonstrate a higher level of knowledge regarding specific techniques due to greater clinical exposure?

## Methods

### Participants and sample characterization

The electronic survey link was distributed exclusively through specialized medical networks and direct invitations to professional associations of gynecologists and obstetricians. This targeted distribution ensured that only individuals meeting the inclusion criteria were reached. Furthermore, the Google Forms® platform was configured to require answers to all questions before submission, ensuring no incomplete datasets. Consequently, 404 participants accessed the link and fully completed the questionnaire, with no exclusions required after data collection.

Inclusion criteria: Licensed Gynecologists-Obstetricians and residents in Gynecology and Obstetrics in Brazil. Exclusion criteria: Non-medical professionals or those who did not complete the questionnaire.

The study included 404 participants, comprising gynecologists-obstetricians (*n* = 361) and residents (*n* = 43). The average age of gynecologists-obstetricians was 48.3 years (SD = 13.4), while the average age of residents was 27.8 years (SD = 2.9). Among the gynecologists and obstetricians, 288 (71.3%) were female, and 73 (18.1%) were male. Among the residents, 39 (9.7%) were female, and 4 (1.0%) were male (see Table 1).

**Table 1:** Participant characteristics (*n* = 404).

Characteristics	Doctors (*n* = 361)	Residents (*n* = 43)
**Age**	48.3 (13.4)	27.8 (2.9)
**Sex**		
Female	288 (71.3%)	39 (9.7%)
Male	73 (18.1%)	4 (1.0%)
**Time of graduation**		
Between 1 and 5 years	32 (7.9%)	42 (10.4%)
Between 6 and 10 years	63 (15.6%)	1 (0.2%)
Between 10 and 20 years	61 (15.1%)	0 (0.0%)
More than 20 years	205 (50.7%)	0 (0.0%)

The professional background of the participants was assessed to establish a baseline for their awareness of specialty society recommendations and previous medical training in FGCS. In this sample, 53.0% (*n* = 214) were unaware of official guidelines from their medical societies, and 76.5% (*n* = 309) had not received formal training or participated in specific discussions on the topic during their residency or medical graduation. These variables provided the technical context for the subsequent analysis of their ethical perceptions and specific knowledge.

### Procedures

The study was approved by the Ethics Committee of the Federal University of Alagoas, Brazil (CAAE: 51752321.3.0000.5013). Data were collected via Google Forms® (Google LLC, Mountain View, CA, USA) between September 24, 2023, and January 11, 2024, using convenience sampling. Participation was anonymous and voluntary; invitations were shared through researchers’ social media and email, without access to participants’ identifying information. All participants were required to access and sign an electronic Free, Prior, and Informed Consent (FPIC) form before starting the questionnaire. Only those who provided consent were included, and participants could withdraw at any time.

### Instrument

The questionnaire consisted of 27 questions and was divided into several sections to collect data on different aspects of professional perception. It began with Participant Characterization, collecting demographic, and professional information such as age, biological sex, religion, years since medical graduation, professional status, and state of residence.

To assess the participants’ perspectives, the survey included questions on the ethical aspects of vulvovaginal cosmetic procedures. These questions evaluated their opinions regarding the justification, ethicality, and benefits of these procedures. For these assessments, the questionnaire used a 5-point Likert scale, where 1 indicated “strongly disagree” and 5 indicated “strongly agree” with the statement.

The instrument also assessed participants’ knowledge of specific aesthetic interventions, such as nymphoplasty (labiaplasty), hymenoplasty, clitoroplasty, pubic mound liposuction, labia majora filler, G-spot augmentation, vaginal rejuvenation, and vulvar whitening. Furthermore, the survey included a section, Patients’ Knowledge About Vulvovaginal Cosmetic Procedures, that evaluated professionals’ perceptions of their patients’ knowledge and concerns, and the influence of sexual partners in patients’ decisions. The instrument also asked participants whether they had performed or undergone any of these procedures and whether they were familiar with their professional societies’ recommendations regarding the indications for these procedures.

### Data analysis

Statistical analysis was performed using R software (version 4.3.2; R Foundation for Statistical Computing, Vienna, Austria). Descriptive statistics were used to characterize the sample and the sociodemographic data.

The normality of the data distribution was verified using the Shapiro–Wilk, Kolmogorov–Smirnov, and Anderson-Darling tests, all of which indicated a non-normal distribution. Consequently, for the comparison between the physician and resident groups, a non-parametric test such as the Mann–Whitney U test was chosen. For the analysis of categorical variables, chi-square tests were used. The statistical significance level was set at *P* < .05.

## Results

The survey included questions on demographic and professional data (age, gender, religion, years since graduation, professional status, and practice location). The main section assessed ethical perspectives on aesthetic procedures—such as labiaplasty, hymenoplasty, and clitoroplasty—using a five-point Likert scale, exploring justification, ethicality, perceived benefits, and knowledge. Another section examined participants’ views on patient motivations, understanding, and partner influence, offering insight into socio-cultural dynamics.

Descriptive statistics (means, SDs, frequencies, and percentages) were used to summarize data. The Shapiro–Wilk, Kolmogorov–Smirnov, and Anderson-Darling tests indicated non-normal distribution (*P* < .001), so the Mann–Whitney U test was used to compare continuous variables between physicians and residents. Chi-square tests were used for categorical variables. The significance level was set at *P* < .05.

The comparative analysis of ethical perspectives and specific knowledge regarding FGCS between doctors and residents. Both groups generally agreed that it is justifiable to use aesthetic procedures to alter the appearance of the vulva or vagina. Doctors reported a mean score of 3.7 (SD = 1.2), while residents had a slightly higher mean score of 3.8 (SD = 1.1). The rank biserial correlation of 0.05 suggests a tiny effect size, with no meaningful difference between the groups (*P* = .577). Both groups rated the ethicality of these procedures similarly, with doctors and residents both scoring a mean of 3.9 (SD = 1.1). The rank biserial correlation of 0.03 indicates minimal difference between the groups (*P* = .772) (see Table 2 for details of the comparison). In terms of perceived health benefits, doctors had a mean score of 3.7 (SD = 1.1), while residents scored slightly lower at 3.6 (SD = 1.1). The rank biserial of −0.02 again shows no notable difference between the groups (*P* = .805). Doctors demonstrated significantly higher knowledge about labiaplasty compared to residents, with a mean score of 4.3 (SD = 0.8) versus 3.6 (SD = 1.1). The rank biserial correlation of −0.39 indicates a moderate effect size (*P* < .001). Doctors also showed greater knowledge about hymenoplasty, scoring a mean of 3.2 (SD = 1.1) compared to residents’ mean of 2.6 (SD = 1.0). The rank biserial correlation of −0.27 suggests a small to moderate difference between the groups (*P* = .003). The knowledge gap was smaller for clitoroplasty, with doctors scoring 2.9 (SD = 1.1) and residents 2.7 (SD = 1.0). The rank biserial of -0.11 indicates a small effect size, with no substantial difference between the groups (*P* = .225). Doctors had more knowledge about pubic mound liposuction, with a mean score of 2.9 (SD = 1.2) compared to 2.5 (SD = 1.1) for residents. The rank biserial of −0.18 suggests a small difference (*P* = .045). Doctors scored higher on knowledge about large labia augmentation, with a mean of 3.2 (SD = 1.1) versus 2.6 (SD = 1.2) for residents. The biserial rank of −0.27 indicates a small to moderate difference (*P* = .003). There was a notable difference in knowledge about G-spot enhancement procedures, with doctors scoring 1.8 (SD = 1.1) and residents scoring 1.3 (SD = 0.6). The rank biserial of −0.22 suggests a moderate difference (*P* = .007). For intimate rejuvenation procedures (CO_2_ laser or radiofrequency), doctors and residents had similar levels of knowledge, with doctors scoring 3.6 (SD = 1.1) and residents 3.5 (SD = 0.9). The rank biserial of −0.09 indicates a small effect size, with no meaningful difference (*P* = .312). Doctors had more knowledge about vulvar whitening procedures, scoring 3.4 (SD = 1.1) compared to residents’ 2.9 (SD = 0.9). The rank biserial of −0.23 suggests a small to moderate difference (*P* = .007). These findings indicate that while both doctors and residents generally agree on the ethicality and justifiability of aesthetic procedures, medical professionals possess significantly more knowledge about specific procedures, likely due to their greater clinical experience. The differences in knowledge between doctors and residents were most pronounced in areas like labiaplasty and hymenoplasty, while the gap was narrower for procedures like clitoroplasty and intimate rejuvenation.

**Table 2:** Comparison of mean ± SD for questions on ethics and specific knowledge in female genital cosmetic surgery.

Questions	Doctors	Residents	Rank biserial	*P*-value^a^
Item 9—Do you consider it justifiable to use aesthetic procedures to alter the appearance of the vulva/vagina?	3.7 ± 1.2	3.8 ± 1.1	0.05	.577
Item 10—Do you consider it ethical to use aesthetic procedures to alter the appearance of the vulva/vagina?	3.9 ± 1.1	3.9 ± 1.1	0.03	.772
Item 11—Do you consider it beneficial for health to use aesthetic procedures to alter the appearance of the vulva/vagina?	3.7 ± 1.1	3.6 ± 1.1	−0.02	.805
Item 12—How much do you know about labiaplasty?	4.3 ± 0.8	3.6 ± 1.1	−0.39	<.001
Item 13—How much do you know about hymenoplasty?	3.2 ± 1.1	2.6 ± 1.0	−0.27	.003
Item 14—How much do you know about clitoroplasty?	2.9 ± 1.1	2.7 ± 1.0	−0.11	.225
Item 15—How much do you know about pubic mound liposuction?	2.9 ± 1.2	2.5 ± 1.1	−0.18	.045
Item 16—How much do you know about large labia augmentation?	3.2 ± 1.1	2.6 ± 1.2	−0.27	.003
Item 17—How much do you know about G-spot enhancement procedures?	1.8 ± 1.1	1.3 ± 0.6	−0.22	.007
Item 18—How much do you know about intimate rejuvenation procedures (CO_2_ laser or radiofrequency)?	3.6 ± 1.1	3.5 ± 0.9	−0.09	.312
Item 19—How much do you know about vulvar whitening procedures?	3.4 ± 1.1	2.9 ± 0.9	−0.23	.007

^a^Mann–Whitney U test.

In the survey, 54.6% of doctors (*n* = 197) and 72.1% of residents (*n* = 31) reported not having undergone any aesthetic vulvovaginal procedures and no desire to do so, comprising 56.4% (*n* = 228) of the total. A small percentage of doctors (1.7%, *n* = 6) preferred not to answer, while no residents selected this option. Additionally, 19.7% of doctors (*n* = 71) and 9.3% of residents (*n* = 4) said the question was not applicable, totaling 18.6% (*n* = 75). Meanwhile, 16.9% of doctors (*n* = 61) and 16.3% of residents (*n* = 7) expressed future interest in such procedures (16.8%, *n* = 68 overall). Lastly, 7.2% of doctors (*n* = 26) and 2.3% of residents (*n* = 1) had already undergone such procedures (6.7%, *n* = 27). The chi-squared test yielded a *P*-value of .171, indicating no significant difference between groups (see Table 3 for details).

**Table 3:** Comparison of response frequency for doctors and residents.

Questions	Doctor (*n* = 361)	Residents (*n* = 43)	Total (*n* = 404)	*P*-value
**Item 20**—Have you ever undergone any procedure (any mentioned in the previous questions or others) for aesthetic purposes in the vulvovaginal region?				.171^a^
No, and I have no desire to do so	197 (54.6%)	31 (72.1%)	228 (56.4%)	
I prefer not to answer	6 (1.7%)	0 (0.0%)	6 (1.5%)	
Not applicable	71 (19.7%)	4 (9.3%)	75 (18.6%)	
No, but I have the desire to do so	61(16.9%)	7 (16.3%)	68 (16.8%)	
Yes	26 (7.2%)	1 (2.3%)	27 (6.7%)	
**Item 21**—Have you ever performed any procedure (any mentioned in the previous questions or others) for aesthetic purposes in the vulvovaginal region on your patients?				.004^b^
No	171 (47.4%)	31 (72.1%)	202 (50.0%)	
Yes	190 (52.6%)	12 (27.9%)	202 (50.0%)	
**Item 22**—If you answered “yes” to the previous question: how many genital aesthetic procedures have you performed?				.007^a^
1	12 (3.3%)	4 (9.3%)	16 (4.0%)	
2	24 (6.6%)	3 (7.0%)	27 (6.7%)	
3	16 (4.4%)	0 (0.0%)	16 (4.0%)	
4	19 (5.3%)	2 (4.7%)	21 (5.2%)	
5 or more	114 (31.6%)	3 (7.0%)	117 (29.0%)	
I prefer not to answer	32 (8.9%)	5 (11.6%)	37 (9.2%)	
None	144 (39.9%)	26 (60.5%)	170 (42.1%)	
**Item 23**—Are you aware of the recommendations from your specialty society regarding the indications for these procedures?				.061^a^
No	184 (51.0%)	30 (69.8%)	214 (53.0%)	
I prefer not to answer	3 (0.8%)	0 (0.0%)	3 (0.7%)	
Yes	174 (48.2%)	13 (30.2%)	187 (46.3%)	
**Item 24**—During your medical training, did you participate in discussions or have a course that addressed the indications for these procedures?				.830^a^
No	276 (76.5%)	33 (76.7%)	309 (76.5%)	
I don’t recall	15 (4.2%)	1 (2.3%)	16 (4.0%)	
Yes	70 (19.4%)	9 (20.9%)	79 (19.6%)	
**Item 25**—Do your patients report dissatisfaction or discomfort with the appearance of their vulvas?				.444^a^
No	82 (22.7%)	7 (16.3%)	89 (22.0%)	
I prefer not to answer	5 (1.4%)	0 (0.0%)	5 (1.2%)	
Yes	274 (75.9%)	36 (83.7%)	310 (76.7%)	

a Pearson’s Chi-squared test.

b Chi-squared test with continuity correction.

Nearly half of the doctors (47.4%, *n* = 171) and most residents (72.1%, *n* = 31) reported that they had never performed aesthetic procedures on their patients, comprising 50.0% (*n* = 202) of the total. Conversely, 52.6% of doctors (*n* = 190) and 27.9% of residents (*n* = 12) indicated that they had performed such procedures, accounting for the other 50.0% (*n* = 202) of the sample. The *P*-value for this comparison was .004, suggesting a difference, with doctors being more likely to have performed these procedures.

Among those who had performed genital aesthetic procedures, 3.3% of doctors (*n* = 12) and 9.3% of residents (*n* = 4) reported performing one procedure, totaling 4.0% (*n* = 16) of the sample. Additionally, 6.6% of doctors (*n* = 24) and 7.0% of residents (*n* = 3) had performed two procedures, making up 6.7% (*n* = 27) of participants. Meanwhile, 4.4% of doctors (*n* = 16) and no residents reported performing three procedures, and 5.3% of doctors (*n* = 19) and 4.7% of residents (*n* = 2) had performed four procedures, accounting for 4.0% (*n* = 16) and 5.2% (*n* = 21) of the sample, respectively. Notably, 31.6% of doctors (*n* = 114) and 7.0% of residents (*n* = 3) had performed five or more procedures, making up 29.0% (*n* = 117) of the total. Some participants, 8.9% of doctors (*n* = 32) and 11.6% of residents (*n* = 5), chose not to disclose the number of procedures performed, contributing to 9.2% (*n* = 37) of the sample. 39.9% of doctors (*n* = 144) and 60.5% of residents (*n* = 26) indicated that they had not performed any procedures, accounting for 42.1% (*n* = 170) of the total. The chi-squared test for this item yielded a *P*-value of .007, indicating differences between doctors and residents in the number of procedures performed.

Lastly, regarding patient reports of dissatisfaction or discomfort with the appearance of their vulvas, 22.7% of doctors (*n* = 82) and 16.3% of residents (*n* = 7) indicated that their patients had not reported such concerns, making up 22.0% (*n* = 89) of the total sample. A small percentage of doctors (1.4%, *n* = 5) and no residents chose not to answer, accounting for 1.2% (*n* = 5) of participants. However, the majority of doctors (75.9%, *n* = 274) and residents (83.7%, *n* = 36) reported that their patients had expressed dissatisfaction or discomfort, contributing to 76.7% (*n* = 310) of the total sample. The *P*-value for this comparison was .444, indicating no difference between doctors and residents regarding patient reports of dissatisfaction or discomfort.

## Discussion

The growing demand for FGCS raises critical questions about professional perception and ethical knowledge in the field of gynecology and obstetrics. Our findings reveal that, while the majority of professionals consider these procedures justifiable and ethical, there is a notable knowledge gap between more experienced physicians and residents. It is important to differentiate between “knowledge”—assessed through specific procedural items—and “formal training”—measured by participant exposure to structured discussions or courses during medical education. Knowledge about specific procedures, such as labiaplasty and hymenoplasty, appears to be acquired primarily in clinical practice rather than through a formal, structured curriculum. It is noteworthy, as highlighted by our results, that more than half of the physicians have performed these surgeries despite the majority reporting a lack of formal training during their residency or specialized courses. This discrepancy is concerning and underscores a potential risk to patient safety, as practitioners may be relying solely on informal learning or practical experience without the support of a structured ethical and technical curriculum. This difficulty in training exposes a critical scenario, as the negative aspects of these procedures tend to manifest in practice. This reinforces the urgent need to integrate structured discussions on the topic into medical curricula to prepare future professionals to navigate the complexity of this landscape in an ethical and informed manner.^10^^,^^16–19^

The inconsistency in formal education raises concerns given a growing demand for FGCS, especially among adolescents and young women, who may make decisions about these interventions without the necessary emotional maturity or adequate knowledge. This reinforces the need for professionals to be able to identify underlying motivations, which are often based on social pressures and distorted perceptions of beauty, and may raise serious ethical questions.

Another important aspect for reflection concerns the commercialization of this procedure as a solution to socially constructed norms, raising significant ethical questions. This is especially concerning in the case of adolescents and young women, who may lack the emotional maturity or knowledge necessary to make fully informed decisions about irreversible body modifications.^20^ As Boddy suggests, this demand is often rooted in deeply ingrained social pressures, making it essential for medical professionals to be equipped with the knowledge and skills necessary to navigate this complex ethical landscape.

In the present study, no significant differences were found between physicians and residents regarding the justification, ethical perspective, and perceived benefits of labiaplasty. The scale used was a 5-point Likert measure, with an observed mean value of approximately 3.7 in both groups. This indicates a perception that the procedure is justifiable or presents meaningful benefits to the patient.

The literature suggests that women’s sexual satisfaction following cosmetic surgical procedures, even in non-genital areas, may be related to an improvement in self-confidence and self-image. The study by Goodman et al. observed a sustained reduction in symptoms of body dissatisfaction after genital surgery, which may indicate that these women suffer from body dissatisfaction rather than true dysmorphia. Although that study did not evaluate the psychiatric function of the sample, other studies with surgical patients in similar populations found results within the normal range.

Nevertheless, even with the seemingly positive surgical outcomes, the literature emphasizes the crucial importance of careful counseling and screening of women seeking female genital aesthetic surgery. The perceived improvement in satisfaction and self-confidence does not diminish the need to evaluate underlying mental health, a crucial point for ethical practice in gynecology.

However, despite reports indicating the low risks associated with these procedures, some discussions highlight potential concerns, particularly in post-surgical contexts where complications such as asymmetry or dehiscence may arise in higher-risk cases, potentially affecting patient outcomes.^20^^,^^21^

The discussion of intimate aesthetic procedures is directly tied to an ethical perspective, as physicians must assess each case individually and determine whether the patient’s genital anatomy is causing physical or psychological distress. If there is an indication that the patient’s motivation is primarily psychological, it is crucial for the physician to recommend psychological counseling and to thoroughly explain the potential risks involved. This allows the patient to make a more informed and secure decision regarding the procedure. In doing so, physicians adhere to ethical principles that guide healthcare practice.^22^

Our study revealed that the majority of doctors and residents (76.7%) reported that their patients had expressed dissatisfaction or discomfort with the appearance of their vulvas. This high rate of preoperative dissatisfaction (as perceived by professionals) contrasts with most studies in the literature, which frequently report high rates of patient satisfaction after labiaplasty, ranging from 90% to 95%.^9^ This discrepancy suggests that the professional’s perception may not directly reflect the patient’s subjective experience after the procedure. The reasons for this difference may lie in the fact that satisfaction studies are frequently based on retrospective self-reports and, in many cases, do not evaluate long-term satisfaction or the presence of social desirability bias. In contrast, the perception of professionals, as captured in our study, may reflect the complexities of clinical practice, including the difficulty of managing patients’ preoperative expectations.

In summary, our findings demonstrate that, while doctors and residents hold a consistent ethical perception regarding the justification of FGCS, there is a critical gap in formal education on the topic. Knowledge appears to be acquired primarily through practical experience, which may not be sufficient to prepare professionals to deal with the complexity of patient motivations and the ethical implications. This research, by providing insight into the perspective of Brazilian professionals, reinforces the need to incorporate structured discussions and ethical training into medical curricula, ensuring that future gynecologists are prepared to offer comprehensive and safe care in an increasingly complex clinical setting.^16^^,^^23^^,^^24^

This study, while providing a valuable contribution to the literature, presents some methodological limitations. First, the use of a cross-sectional study design prevents the establishment of cause-and-effect relationships between variables. It is not possible to determine whether clinical experience leads to greater knowledge, or if greater knowledge leads to greater experience.

Second, the use of a convenience sample and self-report questionnaires may introduce sampling and response biases. Participants’ answers could be influenced by issues related to social desirability. Therefore, it is not always possible to guarantee that the responses represent the true intent of the sample, particularly on more culturally sensitive issues. Additionally, the absence of qualitative questions limits access to the sample’s perception through other methodological strategies. Finally, the study focused on Brazilian professionals, which limits the generalization of the results to other populations.

Future research should focus on a specific sample of physicians who are actively trained and perform these procedures to better evaluate clinical outcomes and the ethical implications within that specialized subset. This approach would complement the current study’s findings on the knowledge and perceptions of the broader specialty.

## Conclusion

This research demonstrates that, while Brazilian healthcare professionals show a consistent ethical perception of FGCS, there is a notable gap in formal knowledge between experienced doctors and residents. Our findings suggest that knowledge about these procedures is acquired primarily through practical experience rather than through a formal, structured curriculum. This discrepancy highlights the urgent need to integrate discussions and training on FGCS into medical curricula, enabling future professionals to navigate the complex ethical landscape of these interventions. Furthermore, given the observed lack of awareness regarding official guidelines, prospective studies should evaluate the specific recommendations of medical societies and investigate how these organizations disseminate such information to ensure it effectively reaches and influences clinical practice. Additionally, the fact that most professionals report their patients expressing dissatisfaction with the appearance of their vulvas contrasts with the literature, which suggests high satisfaction rates. Given this, further investigations should explore the motivations, satisfaction, and potential regrets of patients after labiaplasty to gain a more complete understanding of its real-world impact.
